# Does the *VDR* gene polymorphism influence the efficacy of denosumab therapy in postmenopausal osteoporosis?

**DOI:** 10.3389/fendo.2022.1063762

**Published:** 2023-01-13

**Authors:** Anna Wawrzyniak, Marzena Skrzypczak-Zielińska, Michał Michalak, Marta Kaczmarek-Ryś, Alicja Ewa Ratajczak, Anna Maria Rychter, Kinga Skoracka, Michalina Marcinkowska, Ryszard Słomski, Agnieszka Dobrowolska, Iwona Krela-Kaźmierczak

**Affiliations:** ^1^ Department of Family Medicine, Poznan University of Medical Sciences, Poznań, Poland; ^2^ Institute of Human Genetics, Polish Academy of Sciences, Poznań, Poland; ^3^ Department of Computer Science and Statistics, Poznan University of Medical Sciences, Poznań, Poland; ^4^ Department of Gastroenterology, Dietetics and Internal Diseases, Poznan University of Medical Sciences, Poznań, Poland; ^5^ Doctoral School, Poznan University of Medical Sciences, Poznań, Poland

**Keywords:** *VDR* gene polymorphism, denosumab therapy, postmenopausal osteoporosis, bone fracture, osteoporosis

## Abstract

**Introduction:**

One of the challenges of personalized medicine is a departure from traditional pharmacology toward individualized, genotype-based therapies. Postmenopausal osteoporosis is a prevalent condition requiring intensive treatment, whose effects are measurable only after a long time, and the goal is bone fracture prevention. This study aimed to determine the influence of *VDR* gene variation on anti-osteoporotic one-year treatment with denosumab in 63 Polish women with postmenopausal osteoporosis.

**Materials and methods:**

The correlation between bone mineral density (BMD) of the lumbar vertebral column (L1–L4) and femoral neck, and genotype distributions for the *ApaI*, *BsmI*, *FokI*, and *TaqI* variants of the *VDR* gene was analyzed. Bone fractures during denosumab therapy were also investigated.

**Results:**

In the case of the *Bsml* polymorphism, female patients with BB and Bb genotypes had statistically significantly higher values of BMD and T-score/Z-score indicators, which persisted after a year of denosumab treatment. Our results indicated that the *Bsml* polymorphism contributes to better bone status, and, consequently, to more efficient biological therapy. The study did not reveal significant differences between changes (delta) in BMD and genotypes for the analyzed *VDR* gene *loci*. In the entire study group, one bone fracture was observed in one patient throughout the yearlong period of denosumab therapy.

**Conclusions:**

BB and Bb genotypes of the *Bsml* polymorphism of the *VDR* gene determine higher DXA parameter values both before and after one-year denosumab therapy in postmenopausal women with osteoporosis.

## Graphical Abstract

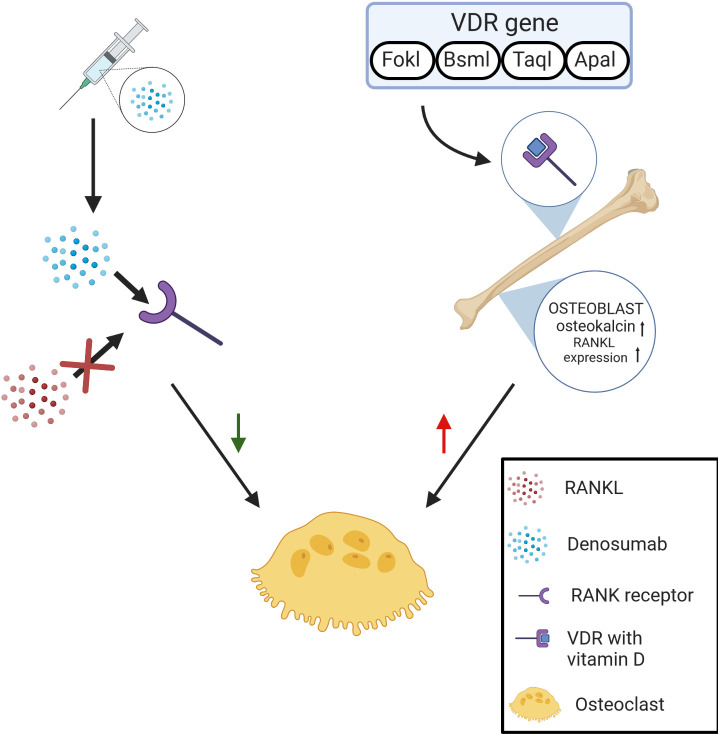

## 1 Introduction

The individualization of treatment is a strategy used in many areas of medicine. In osteoporosis pharmacotherapy, this is extremely important due to hard-to-define therapy objectives and the extensive time it takes to assess treatment. The therapy of osteoporosis - bone disorder, which is a consequence of the imbalance between bone resorption and bone formation - should prevent the first and subsequent osteoporotic fractures. The DXA method indirectly assesses its effects in regular bone mass. Changes in bone mineral density (BMD) reflect the path of transformation, and an increase in density is a substitute indicator of therapy efficacy. The therapy is difficult to manage because DXA assessment makes sense only after one year, at which point a lack of response may also be observed. The fact is that even the most effective drugs do not produce the anticipated results in all patients, and the efficacy of available treatment is evaluated at 70% in terms of vertebral fracture reduction. Therefore, the ability to identify patients - before treatment - who will respond to a drug from those who will not is crucial.

However, pharmacogenetics seems to meet those requirements. Polymorphisms of a number of genes including receptors, e.g. *VDR*, *ESR1*, *PTHR1*, *LRP5*, and *TNFRSF11B* (known as *OPG)* have been described as associated with an increased incidence of fractures and a reduced BMD (1). Therefore, there can be as many candidates for pharmacogenes of osteoporosis as in the case of the polygenic basis of the disease itself. Nevertheless, the *VDR* gene (OMIM 601769), which codes the protein receptor of vitamin D, is one of the best-studied in terms of its association with osteoporosis and bone fractures. Isolated works concern its potential influence on the effect of osteoporosis therapy. Denosumab is a human monoclonal antibody (IgG_2_) that counteracts the receptor activator of nuclear factor kappa beta ligand (RANKL), which blocks its binding with RANK, resulting in an inhibition of osteoclast development and activity. Furthermore, a decrease in bone resorption and an increase in BMD leads to a reduction in the number of vertebral body fractures and non-vertebral fractures (2–5). In osteoblasts 1,25(OH)_2_-D_3_, it increases the synthesis of osteocalcin and osteopontin, as well as ligand expression for osteoprotegerin/RANK (RANKL, *RANK ligand*), which, by binding with its RANK receptor (receptor activator of *NF-*κ *B*) on prosteoclasts, stimulates their maturation into osteoclasts. The common end metabolic pathway within bones, where the activity of osteotropic hormones coincides with many other local factors (including inflammation mediators and immunological responses), is the configuration of osteoprotegerin/RANK-RANKL, which is responsible for osteoclastogenesis and coupling between bone resorption by osteoclasts, and bone formation by osteoblasts (6). Denosumab is used to prevent skeletal-related events, treat Giant cell tumors of the bone, treat hypercalcemia of malignancy, treat osteoporosis, glucocorticoid-induced osteoporosis, and bone loss (7). Denosumab should be considered in contraindication or no effects of standard treatment (8). There is no unified approach to genetic testing in osteoporosis. The only firm conclusion in many meta-analyses is that different populations can differ significantly in this respect (9). Our previous research showed that the *ApaI*, *BsmI*, and *TaqI* polymorphisms of the *VDR* gene could be predictors of low-energy fractures. However, it was not observed that these changes are associated with bone mass (10). The *VDR* gene is located on the long arm of chromosome 12 at position 13.11, has a length of 63,492 base pairs, and consists of 11 exons. VDR protein belongs to the nuclear receptor family and functions as a transcription factor. It was found that the polymorphisms of four *loci* - *ApaI* (rs7975232), *BsmI* (rs1544410), *FokI* (rs10735810), and *TaqI* (rs731236) - within the *VDR* gene are related to changes BMD (11–13). The literature contains no studies indicating a correlation between denosumab therapy efficacy and *VDR* gene polymorphism. However, it was proven that the optimal (at least 50 nmol/L) concentration of 25 (OH) vitamin D in serum, as a ligand of the VDR receptor, improves denosumab therapy efficacy. This observation is still being explained (14). Pharmacogenetic studies raise the question of whether polymorphism of the *VDR* gene influences denosumab treatment efficacy in women with postmenopausal osteoporosis. The objective of this study was to answer this question by assessing BMD changes in the lumbar spine (L1–L4) and femoral neck over one year, as well as the incidence of new fractures during therapy in female patients with single *ApaI*, *BsmI*, *FokI* and *TaqI* polymorphic variations of the *VDR* gene.

This study aimed to determine the influence of *VDR* gene variants on anti-osteoporotic one-year treatment with denosumab in 63 Polish women with postmenopausal osteoporosis.

## 2 Materials and methods

The sample group consisted of 63 women, aged between 57 and 86 (mean 75.0 ± 7.3 years) being treated for postmenopausal osteoporosis from Greater Poland in the Endocrinology and Osteoporosis Outpatient Clinic [Poznan University of Medical Sciences, Heliodor Swiecicki Hospital]. In the group of women enrolled in the study before denosumab therapy began, 55 (87.3%) had been diagnosed with bone fractures, including 40 (63.49%) with vertebral fractures, 6 (9.52%) with femoral neck fractures, and 36 (57.14%) with non-vertebral fractures. The patients were subject to one year of observation while undergoing denosumab therapy in the form of single, 60 mg subcutaneous injections of denosumab once every six months, in accordance with the product profile (2, 15, 16). All patients used 800–1,200 mg/day of standard calcium supplementation and up to 1,000 IU/day of vitamin D. All patients gave their written consent to participate in the study and genetic testing. The study was approved by the Bioethical Committee of the Poznan University of Medical Sciences (Poland; approval no. 508/13).

The inclusion criteria were diagnosis of postmenopausal osteoporosis according to the WHO criteria and lack of contraindications to denosumab treatment in accordance with the product profile. Based on medical history, clinical examination, and laboratory tests, women with secondary variants of the disease (i.e. hyperthyroidism, hyperparathyroidism, Cushing’s disease, kidney disease, rheumatoid arthritis, malabsorption syndromes) were excluded from the group. To investigate the associations of single *ApaI*, *BsmI*, *FokI*, and *TaqI* polymorphic variants of the *VDR* gene with changes in BMD over one year, molecular and densitometry tests using the dual-photon X-ray absorptiometry (DXA) method were conducted twice – once before and once after the one-year denosumab therapy period using the DPX-Plus device (Lunar). The incidence of bone fractures during and after the one-year denosumab treatment period was also assessed.

### 2.1 Genotyping

DNA was isolated from peripheral blood leukocytes by the guanidinium isothiocyanate method (17). The polymerase chain reaction (PCR) was carried out in 20 μl with 200 ng genomic DNA, 50 mM KCl, 10 mM Tris–HCl (pH 8.3), 1.5 mM MgCl_2_, 0.25 mM dNTP, 7.5 pmol of each primer, and 0.5 units of Taq polymerase (Sigma). The reaction was conducted as follows: initial denaturation at 94°C for 4 min; followed by 35 cycles of denaturation at 94°C or 40 s; primer annealing for 40 s; elongation at 72°C for 100 s; final incubation at 72°C for 180 s. For *BsmI* polymorphism, 837 bp fragment was amplified at an annealing temperature of 55°C using primers forward 5’-GGCAACCAAGACTACAAGTACC-3’ and reverse 5’-TCTTCCACCTCTAACCAGCG-3’. For *FokI*, 267 fragment was amplified at an annealing temperature of 60°C using primers forward 5’- AGCTGGCCCTGGCACTGACTCTGCTCT -3’ and reverse 5’- ATGGAAACACCTTGCTTCTTCTCCCTC-3’ (18, 19). For *ApaI* and *TaqI* polymorphisms of the *VDR* gene, 745 bp fragment was amplified at an annealing temperature of 64°C using primers forward 5’-CAG AGCATGGACAGGGAGCAA-3’ and reverse 5’-GCACTCCTCATGGCTGAGGTCTC-3’ (19). The PCR product was then subjected to restriction fragment length polymorphism (RFLP) analysis using the following restriction enzymes: TaqI (Fermentas); ApaI, Bsp120I (Fermentas); BsmI, and Mva1269I (Fermentas). PCR and RFLP analysis was performed using Applied Biosystems 2720 Thermal Cycler (Applied Biosystems, Foster City, CA). All analyses were carried out according to the manufacturer’s recommendations, and the products of hydrolysis were separated on 1.5% agarose gel and visualized with ethidium bromide.

The numbers of single alleles were calculated as a sum of a double number of homozygotes (dominant or recessive) and a single number of heterozygotes of each studied allele.

### 2.2 Statistical analysis

Before starting this study, we preliminarily assumed that during the follow-up (one year), we might expect an increase of BMD by 5% and changes in T-score and Z-score by 20%. Taking this assumption and the significance level at α = 0.05 and the power of the test as 80%, we have calculated a minimal sample size of n = 21 for BMD and n = 43 for T- and Z-scores. Finally, we took the sample size n = 63 since that number of patients consented to the study.

All data are expressed as the mean ± SD, unless otherwise stated. The analyzed data were expressed on an interval and nominal scale. To compare the two groups, Student’s t test for dependent data was performed, or, in the absence of the required assumptions (normality and homogeneity of variance), the Wilcoxon test was used. The calculation regarding normal distribution was checked by Shapiro-Wilks test. The Chi-square test or Fisher exact test was used to analyze nominal data. When comparing more than two groups simultaneously, a univariate analysis of variance with Tukey *post hoc* test was used. Additionally, we checked the Hardy-Weinberg equation for analyzed polymorphisms to measure whether the observed genotype frequencies in a studied population differ from the frequencies predicted by the equation. All tests were analyzed at the significance level of α = 0.05. Statistical analysis was done using Statistica 13.0 software (Stat Soft Inc, Tulsa, USA) and STATA 15.1 (StataCorp LLC).

#### 3 Results

**Figure 1:**
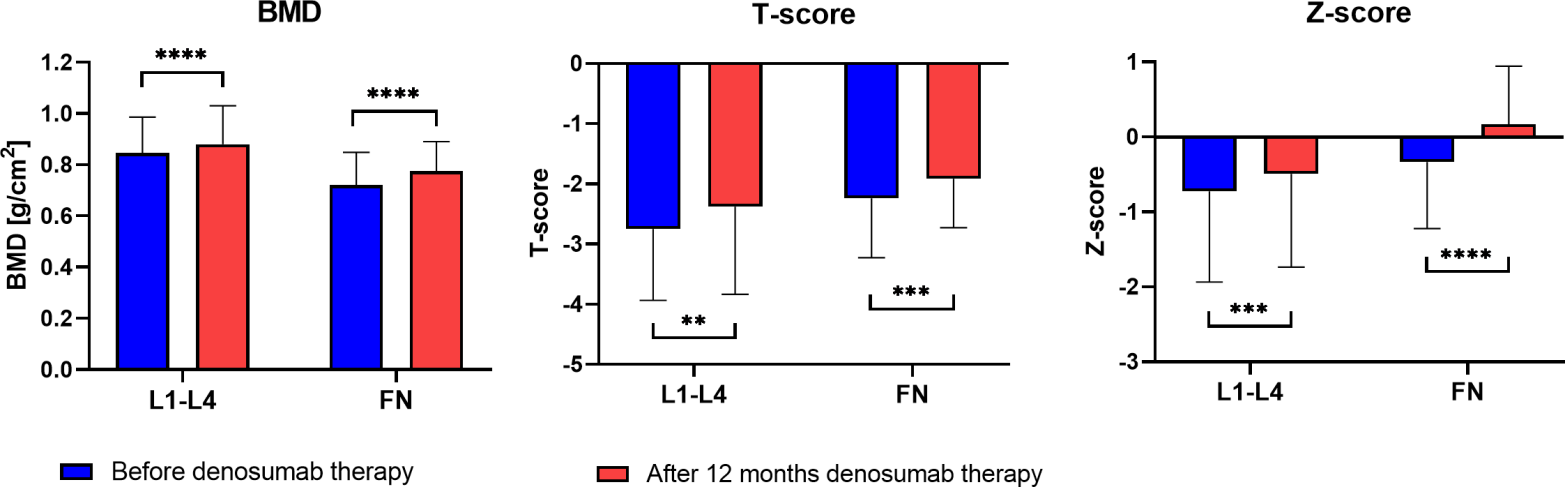
DXA parameters of the lumbar spine and femoral neck (FN)) before and after one year of denosumab therapy in the sample group (**P < 0.01, ***P < 0.001, ****P < 0.001).

The mean BMI value in the sample group was 23.9 ± 2.9 kg/m^2^ (mean body weight: 58.3 ± 7.4 kg, mean height: 154.3 ± 6.7 cm). The time since menopause occurs, on average, is 25.8 ± 8.5 years. The mean baseline level of 25-hydroxyvitamin D in the study group was 25.2 ± 7.1 ng/ml. The average values of DXA measurements of the lumbar vertebral column (L1–L4) and femoral neck prior to biological treatment and one year after denosumab therapy are presented in **Figure 1** and **Supplementary Table S1**, **2**.

The incidence of bone fractures was also observed in the sample group. During the entire denosumab treatment period (12 months), one event of vertebral fractures was observed in one female patient. Due to low variability, this was not subject to statistical analysis.

In the case of all 4 *loci*, both types of homozygotes (AA, aa, BB, bb, FF, ff, TT and tt) and heterozygotes (Aa, Bb, Ff and Tt) were observed. The *TaqI* polymorphism (T allele 66.94% and t allele 33.06%) showed the lowest variability. For the remaining changes, the rate of alleles was closer to 50% (**Table 1**). In contrast to *ApaI* and *TaqI* polymorphisms, *Bsml* and *Fokl* polymorphisms were not in accordance with the Hardy–Weinberg principle (*P* > 0.05), **Table 1**.

**Table 1:** Allele and genotype distribution for the *ApaI*, *BsmI*, *FokI* and *TaqI* polymorphisms of the *VDR* gene in the sample group.

*VDR* change	Frequency					HWE	
	Genotypes			Alleles		χ^2^ (df = 1)	*P*
ApaI *n* = 63	AA	Aa	aa	A	a	4.926	0.0265
	*n* = 15 (23.81%)	*n* = 22 (34.92%)	*n* = 26 (41.27%)	*n* = 52 (41.27%)	*n* = 74 (58.73%)		
BsmI *n* = 63	BB	Bb	bb	B	b	1.162	0.2811
	*n* = 28 (44.44%)	*n* = 25 (39.69%)	*n* = 10 (15.87%)	*n* = 81 (64.29%)	*n* = 45 (35.71%)		
FokI *n* = 63	FF	Ff	ff	F	f	0.169	0.6806
	*n* = 13 (20.63%)	*n* = 33 (52.38%)	*n* = 17 (26.98%)	*n* = 59 (46.83%)	*n* = 67 (53.17%)		
TaqI *n* = 62	TT	Tt	tt	T	t	5.869	0.0154
	*n* = 32 (51.61%)	*n* = 19 (30.65%)	*n* = 11 (17.74%)	*n* = 83 (66.94%)	*n* = 41 (33.06%)		

n, number of patients; HWE, Hardy-Weinberg Equilibrium.

**Figure 2:**
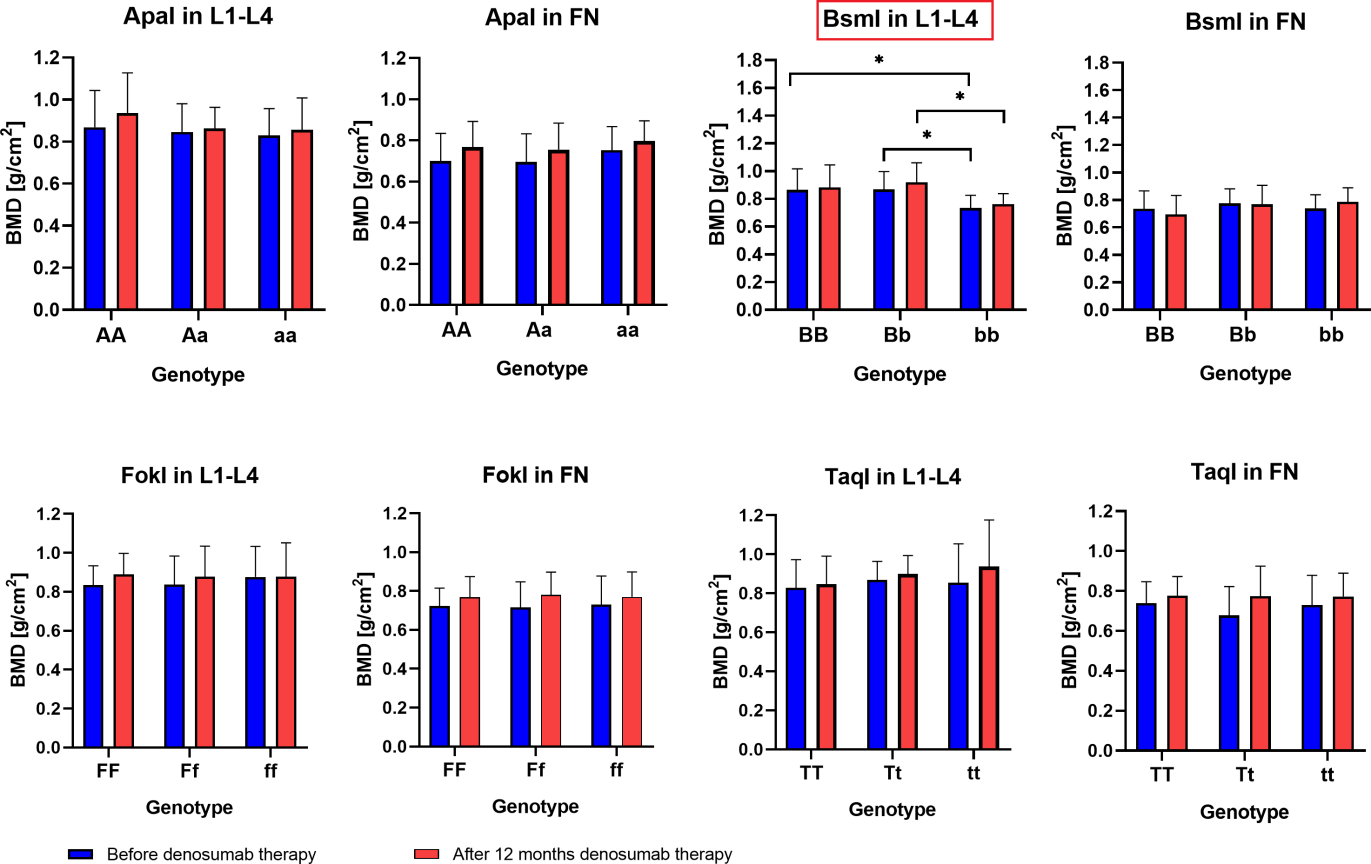
BMD results in the femoral neck (FN) and L1–L4 area for *ApaI*, *BsmI*, *FokI*, and *TaqI* genotypes of the *VDR* gene in a studied group before and after one year of denosumab therapy (*P < 0.05).

**Figure 3:**
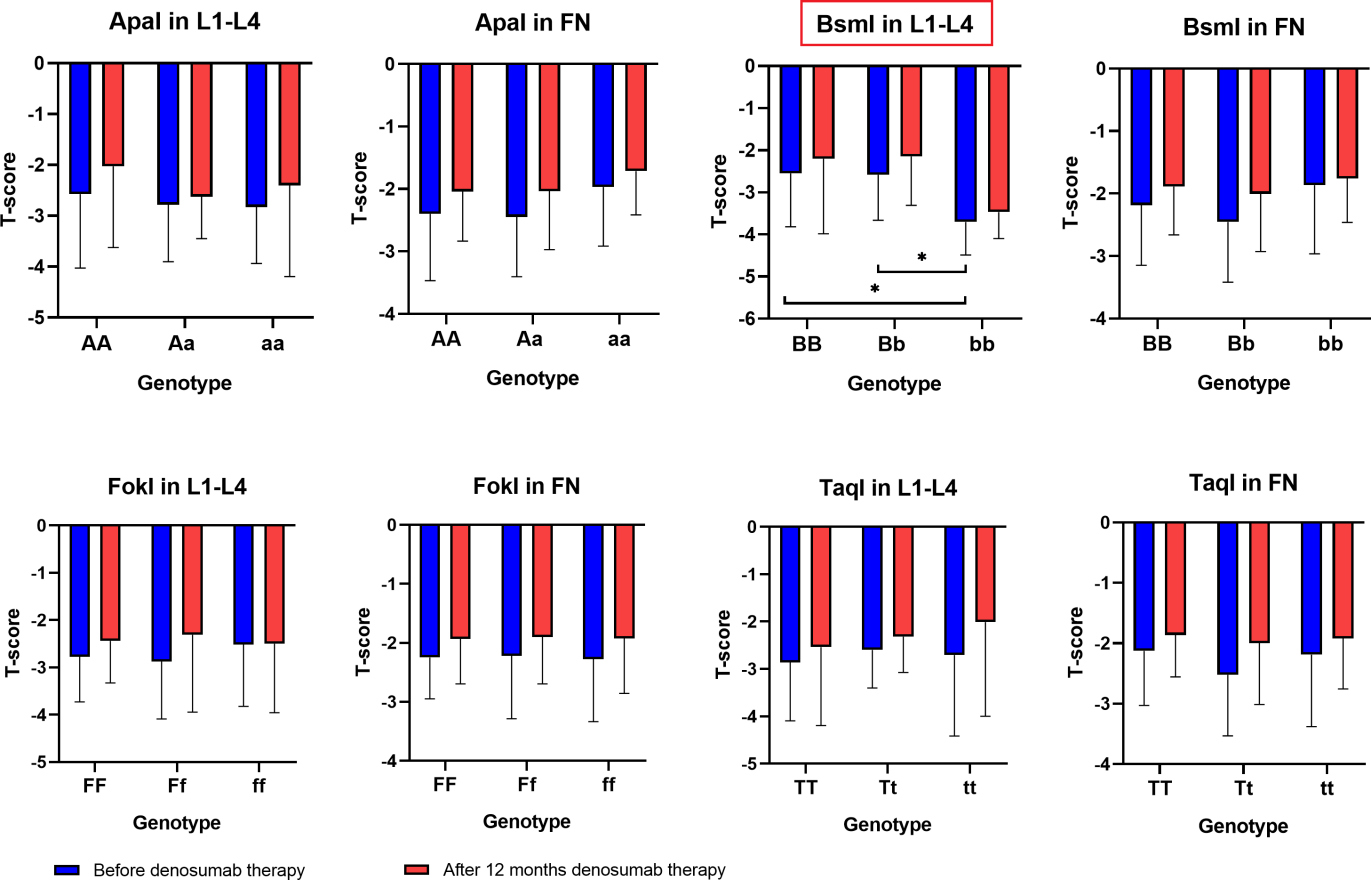
T-score results in the femoral neck (FN) and L1–L4 area for *ApaI*, *BsmI*, *FokI*, and *TaqI* genotypes of the *VDR* gene in a studied group before and after one year of denosumab therapy (*P < 0.05).

**Figure 4:**
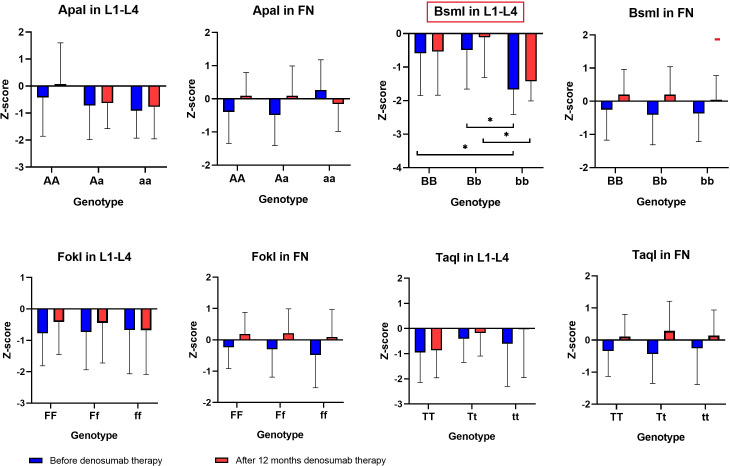
Z-score results in the femoral neck and L1–L4 area for *ApaI*, *BsmI*, *FokI*, and *TaqI* genotypes of the *VDR* gene in a studied group before and after one year of denosumab therapy (*P < 0.05).

The results of BMD, T-score and Z-score before and after one year of denosumab therapy in the area of the femoral neck and L1–L4 for genotypes the *ApaI*, *BsmI*, *FokI*, and *TaqI* of the *VDR* gene are illustrated in **Figures 2**–**4**. Moreover, the calculated differences between delta changes in densitometry parameters are presented in **Supplementary Tables S3**-**S9** and in **Table 2** (due to statistically significant results).

**Table 2:** Delta changes in DXA parameters for the *VDR* BsmI polymorphism in L1-L4.

Results of DXA (L1-L4)	Genotype			*P* value
	BB *n* = 28	Bb *n* = 25	bb *n* = 10	^*^BB vs Bb ^**^BB *vs* bb ^***^Bb *vs* bb
BMD I	0.865 ± 0.151	0.868 ± 0.129	0.734 ± 0.091	*P* = 0.0215 ** p = 0.026 *** p = 0.028
BMD II	0.883 ± 0.162	0.919 ± 0.141	0.763 ± 0.074	*P* = 0.0394 *** *P* = 0.030
BMD (g/cm^2^) Δ	0.039 ± 0.074	0.0540 ± 0.056	0.031 ± 0.052	ns
T-score I	-2.549 ± 1.27	-2.585 ± 1.076	-3.700 ± 0.785	*P* = 0.0191 ** *P* = 0.021 *** *P* = 0.028
T-score II	-2.205 ± 1.776	-2.150 ± 1.157	-3.469 ± 0.631	ns
T-score SD Δ	0.509 ± 1.504	0.460 ± 0.453	0.231 ± 0.407	ns
Z-score I	-0.587 ± 1.262	-0.493 ± 1.161	-1.672 ± 0.742	*P* = 0.0222; ** *P* = 0.022 *** *P* = 0.036
Z-score II	-0.534 ± 1.305	-0.113 ± 1.200	-1.421 ± 0.589	*P* = 0.0342 *** *P* = 0.026
Z-score SD Δ	0.213 ± 0.702	0.387 ± 0.541	0.294 ± 0.396	ns

ns - non significant, P – ANOVA P value; Tukey post-hoc test P values: *BB vs Bb; **BB vs bb; ***Bb vs bb.

It was observed that the BMD of the lumbar vertebral column bones (L1–L4) both before (*P* = 0.0215) and after denosumab treatment (*P* = 0.0394), as well as T-score values (before treatment *P* = 0.0191) and Z-score values (before treatment *P* = 0.0222 and after treatment P = 0.0342), are statistically significantly dependent on the *Bsml* polymorphism (**Table 2**). The bb genotype contributes to lower BMD and T-score/Z-score indicators values, whereas BB and Bb genotypes are characteristic for female patients with better bone DXA parameters. However, the differences (BMD Δ, T score Δ, Z score Δ) calculated from the recorded DXA parameters for lumbar vertebral column bones (L1–L4) and femoral neck bones do not show any significant correlation with genotypes for the *ApaI*, *BsmI*, *FokI*, and *TaqI* polymorphic variants of the *VDR* gene. Therefore, comparing DXA results before and after the sample group’s denosumab treatment indicates an upward trend for single genotypes. This trend is not statistically significant, but the vital differences between single genotypes are preserved.

## 4 Discussion

The molecular diagnostics of osteoporosis aim to identify polymorphisms that determine predispositions to developing this disease. Assessment of its genetic foundations is difficult in the case of a multi-gene conditioned phenotype, which is subject to additional modifications due to the strong influence of many environmental factors. Single polymorphisms of genes involved in bone tissue metabolism insignificantly influence phenotype, and only the combined effect of adverse changes in many genes is visible. Moreover, the phenotype is further affected by the interference of environmental factors such as diet, lifestyle, and hormones. Therefore, a genotype predisposed to osteoporosis is not an indicator of its occurrence. Anticipating responses to the pharmacological treatment given certain genetic conditions is becoming increasingly important. A meta-analysis of 25 studies in which 4,075 postmenopausal women in China were assessed showed a statistically significant relationship between *Apal* and *Bsml* polymorphisms of the *VDR* and BMD genes was observed. BMD was significantly higher in heterozygotes of the polymorphisms studied (20).

This study focuses on the analysis of 4 polymorphisms of one gene, the protein product of which is significantly involved in bone tissue metabolism. The vitamin D receptor (*VDR*) was one of the first genes studied for its influence on osteoporosis development. Moslai et al. observed that vitamin D concentration influences the response to denosumab treatment (14). Building on their assumptions regarding the correlation between bisphosphonate activity and vitamin D concentration, it might also be the case that vitamin D with auto/paracrine properties can be synergically active with denosumab, improving bone mineralization by stimulating osteoblast maturation and mineralization-related gene expression (14, 21). The protein of vitamin D receptors could play an essential role in this process. Given these considerations, we decided to assess four main polymorphisms (*ApaI*, *BsmI*, *FokI*, *TaqI*) of the vitamin D receptor gene for their responses to denosumab treatment. For many years, *VDR* gene polymorphisms have been the subject of global research that has yielded a wide range of results in different populations (9, 10). Seremak-Mrozikiewicz A. et al. showed that the T allele of the *TaqI* polymorphism could determine a higher risk of osteoporosis in postmenopausal women. Consequently, the t allele could have a protective effect. The presence of the A allele (*ApaI* polymorphism) seems to be loosely connected with susceptibility to osteoporosis (22).

Although genetic tests concerning the relation between *VDR* genotypes and bone quality yield contrary results, pharmacogenetic tests on individual responses to osteoporosis treatment yield more consistent and important data. Hormonal replacement therapy (HRT), used in monotherapy or in conjunction with alendronate, results in a significant increase of BMD in postmenopausal women (23, 24). Essentially, the majority of pharmacogenetic studies concentrate on response variability to HRT (25–27). Research on Italian women with postmenopausal osteoporosis addressed the influence of two polymorphisms of the *VDR* gene - *BsmI* and *FokI* - on BMD in response to bisphosphonate treatment or strontium ranelate treatment demonstrated that *FokI*, but not *BsmI*, can influence the response to postmenopausal osteoporosis therapy. This conclusion supports the idea that treatment should be individualized (28).

Denosumab is a fully human monoclonal antibody that counteracts the receptor activator of nuclear factor kappa beta ligand (RANKL). It diminishes the generation, function, and survival of osteoclasts and is used to treat postmenopausal women with osteoporosis with increased or high fracture risk. During the 3-year FREEDOM study, 60 mg of denosumab administered subcutaneously every six months significantly decreased the number of vertebral column fractures (68%), hip fractures (40%), and non-vertebral fractures (20%). It also increased BMD and decreased the activity of bone turnover markers to a greater extent than placebos in postmenopausal women with osteoporosis (29). This research was continued through a comparison of the effects of denosumab in postmenopausal women with osteoporosis over a 5-year period to a placebo group treated for only three years. Five years of therapy decreased the activity of bone turnover markers (BTM) and increased BMD, which was associated with a low number of fractures and a favorable risk-benefit ratio (30).

As in many other investigations, our study revealed that in all women with postmenopausal osteoporosis, one year of denosumab therapy was beneficial and resulted in increased BMD. During the treatment, one new bone fracture was reported in one woman. In randomized studies, 60 mg of denosumab significantly reduced the fracture risk compared to placebo and bisphosphonate treatment, and decreased the bone turnover marker value, while simultaneously increasing the BMD (2, 31–39). Nevertheless, recent observations have shown that discontinuation of denosumab treatment is associated with decreased BMD and bone fracture risk. Popp AW et al. proved that termination of denosumab treatment after long-term exposure resulted in a significant BMD decrease at all measured sites, which suggests that treatment duration can predict the speed and volume of bone loss (40). Moreover, another study observed vertebral fractures after discontinuation of denosumab (41). Therefore, the search for a genetically conditioned response to denosumab treatment therefore appears justified concerning long-term post-treatment.

Our results did not reveal statistically significant differences between delta changes (after one year of denosumab treatment) in densitometric parameters in the area of the femoral neck and L1–L4 for the following single genotypes of the *VDR* gene: AA, Aa and aa *Apa*; BB, Bb and bb *BsmI*; FF, Ff and ff *FokI*; and TT, Tt and tt *TaqI*. However, in the case of the *Bsml* polymorphism, it was interestingly observed that female patients with BB and Bb genotypes have statistically significantly higher values of BMD and T-score/Z-score indicators, which persist after a year of denosumab treatment. We can claim that the *Bsml* polymorphism determines better bone status in female patients, which continues over one year of treatment with denosumab. Though this observation was not the primary objective, it can help guide the denosumab treatment process. Further studies should verify this conclusion by prolonging the observation period and increasing the sample group size. Focusing on studies from recent years regarding the impact of the *BsmI* variant of the *VDR* gene in women with postmenopausal osteoporosis, regardless of the treatment, the results are discrepant. On the one hand, favorable relationships between the bb variants and bone density were observed, which partly confirms the results of our research. Namely, other Polish researchers showed that women with the bb genotype presented lower BMD values of the hip compared to patients with the BB or Bb genotypes (42). In our results, the dependencies concerned the lumbar vertebral column (L1–L4) but indicated a positive impact of ‘BB’ or ‘Bb’ genotypes on DXA parameters. Pederera-Canal et al., after five years of observation of patients with postmenopausal osteoporosis, found a significant modification of BMD at the femoral neck based on the rs1544410 genotype (BB *vs*. Bb) i. e. the BB group gained more BMD than the Bb group (43). On the other hand, many recent studies have not shown a relationship between the *BsmI* polymorphism and bone density in Spanish postmenopausal women (44) or that this variant was related to an increased risk of osteoporosis (45–47).

Moreover, polymorphism studies of genes other than *VDR* genes involved in bone metabolism - especially the metabolic pathway RANKL-RANK-OPG, influenced by the studied drug, denosumab – may prove more accurate for individualized antiosteoporotic treatment. Increasing the sample group size and extending the observation period would be a valuable addition to the study. Furthermore, it is worth considering studies of other genes, especially from the RANKL-RANK-OPG metabolic pathway.

## 5 Conclusions

In the group of 63 Polish women with postmenopausal osteoporosis, BB and Bb genotypes of the *Bsml* polymorphism determine higher DXA parameter values both before and after biological treatment. No statistically significant differences between the *ApaI*, *BsmI*, *FokI*, and *TaqI* polymorphisms of the *VDR* gene in response to one-year denosumab therapy were observed.

## Data availability statement

The original contributions presented in the study are included in the article/**Supplementary Material**. Further inquiries can be directed to the corresponding author.

## Ethics statement

The studies involving human participants were reviewed and approved by Bioethical Committee of the Poznan University of Medical Sciences. The patients/participants provided their written informed consent to participate in this study.

## Author contributions

Conceptualization, AW, MM, and IK-K; methodology, AW, MS-Z, MMi, MK-R, MM, and IK-K; investigation, AW and IK-K; writing—original draft preparation, AW, MS-Z, AER, AMR, and KS; writing—review and editing, RS, AD, and IK-K; visualization, MS-Z; supervision, IK-K; project administration, AD. All authors have read and agreed to the published version of the manuscript.
