# Supplementary material for: Does the VDR gene polymorphism influence the efficacy of denosumab therapy in postmenopausal osteoporosis?
Source: Front Endocrinol (Lausanne). 2023 Jan 13;13:1063762. doi: 10.3389/fendo.2022.1063762 (PMC9880251; doi:10.3389/fendo.2022.1063762)
Supplement: Supplementary file 1 [file Table_1.docx]

Supplementary Table S1. Characteristics of DXA parameters of the lumbar spine (L1-L4) before and after one year of denosumab therapy in the sample group.

| DXA parameters  (L1-L4) | Before  denosumab  therapy | After 12 months  following denosumab therapy | ∆ | *P value* |
| --- | --- | --- | --- | --- |
| BMD (g/cm^2^) I | 0.845 ± 0.141 | 0.880 ± 0.150 | 0.044 ± 0.063 | *P* < 0.0001 |
| T score SD I | -2.749 ± 1.188 | -2.376 ± 1.458 | 0.445 ± 1.024 | *P* = 0.0028 |
| Z score SD I | -0.724 ± 1.211 | -0.493 ± 1.241 | 0.299 ± 0.593 | *P* = 0.0006 |

**Supplementary Table S2.** Characteristics of DXA parameters of the femoral neck (FN)) before and after one year of denosumab therapy in the sample group.

| DXA parameters (FN) | Before  denosumab  therapy | After 12 months  following  denosumab therapy | ∆ | *P value* |  |
| --- | --- | --- | --- | --- | --- |
| BMD (g/cm^2^) I | | 0.720 ± 0.128 | 0.774 ± 0.116 | 0.056 ± 0.079 | *P* < 0.0001 |
| T-score SD I | | -2.240 ± 0.989 | -1.913 ± 0.814 | 0.346 ± 0.666 | *P* = 0.0004 |
| Z-score SD I | | -0.334 ± 0.889 | 0.173 ± 0.773 | 0.542 ± 0.541 | *P* < 0.0001 |

**Supplementary Table S3.** Delta changes in DXA parameters for the *VDR* ApaI polymorphism in L1-L4.

| **Results of DXA  (L1-L4)** | Genotype | | | *P value*  *^*^AA vs Aa*  *^**^AA vs aa*  *^***^Aa vs aa* |
| --- | --- | --- | --- | --- |
|  | AA  *n* = 15 | Aa  *n* = 22 | Aa  *n* = 25 |  |
| **BMD I** | 0.868 ± 0.175 | 0.846 ± 0.134 | 0.830 ± 0.128 | ns |
| BMD II | 0.935 ± 0.192 | 0.862 ± 0.101 | 0.856 ± 0.152 | ns |
| BMD (g/cm^2^)∆ | 0.057 ± 0.064 | 0.046 ± 0.051 | 0.033 ± 0.074 | ns |
| T-score I | -2.574 ± 1.451 | -2.781 ± 1.121 | -2.826 ± 1.109 | ns |
| T-score II | -2.027 ± 1.597 | -2.621 ± 0.826 | -2.401 ± 1.791 | ns |
| T-score SD ∆ | 0.459 ± 0.549 | 0.406 ± 0.417 | 0.472 ± 1.564 | ns |
| Z-score I | -0.420 ± 1.438 | -0.720 ± 1.260 | -0.910 ± 1.019 | ns |
| Z-score II | 0.073 ± 1.53 | -0.628 ± 0.946 | -0.767 ± 1.183 | ns |
| Z-score SD ∆ | 0.394 ± 0.519 | 0.334 ± 0.526 | 0.201 ± 0.702 | ns |

ns - non significant, P – ANOVA P value; Tukey post-hoc test P values: *AA vs Aa; **AA vs aa; ***Aa vs aa

**Supplementary Table S4.** Delta changes in DXA parameters for the *VDR* ApaI polymorphism in femoral neck.

| **Results of DXA**  **(Femoral Neck)** | Genotype | | | *P value*  *^*^AA vs Aa*  *^**^AA vs aa*  *^***^Aa vs aa* |
| --- | --- | --- | --- | --- |
|  | AA  *n* = 15 | Aa  *n* = 22 | Aa  *n* = 25 |  |
| **BMD I** | 0.700 ± 0.134 | 0.696 ± 0.136 | 0.752 ± 0.115 | Ns |
| BMD II | 0.767 ± 0.125 | 0.755 ± 0.129 | 0.797 ± 0.098 | ns |
| BMD (g/cm^2^)∆ | 0.067 ± 0.080 | 0.059 ± 0.08 | 0.047 ± 0.081 | ns |
| T-score I | -2.398 ± 1.071 | -2.449 ± 0.956 | -1.968 ± 0.946 | ns |
| T-score II | -2.043 ± 0.793 | -2.035 ± 0.936 | -1.715 ± 0.700 | ns |
| T-score SD ∆ | 0.355 ± 0.432 | 0.424 ± 0.506 | 0.266 ± 0.912 | ns |
| Z-score I | -0.394 ± 0.953 | -0.489 ± 0.920 | -0.157 ± 0.826 | ns |
| Z-score II | 0.093 ± 0.699 | 0.093 ± 0.898 | 0.308 ± 0.716 | ns |
| Z-score SD ∆ | 0.487 ± 0.424 | 0.635 ± 0.603 | 0.489 ± 0.575 | ns |

ns - non significant, P – ANOVA P value; Tukey post-hoc test P values: *AA vs Aa; **AA vs aa; ***Aa vs aa

**Supplementary Table S5.** Delta changes in DXA parameters for the *VDR* BsmI polymorphism in femoral neck.

| **Results of DXA**  **(Femoral Neck)** | Genotype | | | *P* value  ^*^BB *vs* Bb  ^**^BB *vs* bb  ^***^Bb *vs* bb |
| --- | --- | --- | --- | --- |
|  | BB  *n* = 28 | Bb  *n* = 25 | bb  *n* = 10 |  |
| **BMD I** | 0.735 ± 0.131 | 0.696 ± 0.137 | 0.737 ± 0.100 | ns |
| BMD II | 0.775 ± 0.106 | 0.769 ± 0.137 | 0.787 ± 0.101 | ns |
| BMD (g/cm^2^) ∆ | 0.049 ± 0.076 | 0.072 ± 0.091 | 0.04 ± 0.058 | ns |
| T-score I | -2.186 ± 0.961 | -2.456 ± 0.961 | -1.865 ± 1.100 | ns |
| T-score II | -1.890 ± 0.77 | -2.005 ± 0.925 | -1.759 ± 0.703 | ns |
| T-score SD ∆ | 0.374 ± 0.535 | 0.462 ± 0.474 | 0.002 ± 1.169 | ns |
| Z-score I | -0.255 ± 0.917 | -0.41 ± 0.903 | -0.371 ± 0.844 | ns |
| Z-score II | 0.2 ± 0.757 | 0.2 ± 0.842 | 0.049 ± 0.723 | ns |
| Z-score SD ∆ | 0.499 ± 0.594 | 0.626 ± 0.578 | 0.448 ± 0.24 | ns |

ns - non significant, P – ANOVA P value; Tukey post-hoc test P values: *BB vs Bb; **BB vs bb; ***Bb vs bb

**Supplementary Table S6.** Delta changes in DXA parameters for the *VDR* Fokl polymorphism in L1-L4.

| **Results of DXA**  **(L1-L4)** | Genotype | | | P value  ^*^FF vs Ff  ^**^FF vs ff  ^***^Ff vs ff |
| --- | --- | --- | --- | --- |
|  | FF  *n* = 13 | Ff  *n* = 33 | ff  *n* = 17 |  |
| **BMD I** | 0.834 ± 0.100 | 0.837 ± 0.146 | 0.876 ± 0.157 | ns |
| BMD II | 0.889 ± 0.108 | 0.877 ± 0.157 | 0.877 ± 0.174 | ns |
| BMD (g/cm^2^)∆ | 0.073 ± 0.051 | 0.036 ± 0.063 | 0.040 ± 0.072 | ns |
| T-score I | -2.773 ± 0.955 | -2.874 ± 1.216 | -2.516 ± 1.305 | ns |
| T-score II | -2.440 ± 0.885 | -2.312 ± 1.632 | -2.500 ± 1.455 | ns |
| T-score SD ∆ | -0.420 ± 0.774 | 0.487 ± 1.212 | 0.352 ± 0.616 | ns |
| Z-score I | -0.772 ± 1.038 | -0.732 ± 1.206 | -0.672 ± 1.392 | ns |
| Z-score II | -0.420 ± 1.036 | -0.449 ± 1.274 | -0.682 ± 1.403 | ns |
| Z-score SD ∆ | 0.477 ± 0.727 | 0.215 ± 0.534 | 0.375 ± 0.633 | ns |

ns - non significant, P – ANOVA P value; Tukey post-hoc test P values: *FF vs Ff; **FF vs ff; ***Ff vs ff

**Supplementary Table S7.** Delta changes in DXA parameters for the *VDR* Fokl polymorphism in the femoral neck.

| **Results of DXA**  **(Femoral Neck)** | Genotype | | | P value  *FF vs Ff  **FF vs ff  ***Ff vs ff |
| --- | --- | --- | --- | --- |
|  | FF  *n* = 13 | Ff  *n* = 33 | ff  *n* = 17 |  |
| **BMD I** | 0.722 ± 0.094 | 0.715 ± 0.132 | 0.729 ± 0.148 | ns |
| BMD II | 0.768 ± 0.106 | 0.78 ± 0.117 | 0.769 ± 0.129 | ns |
| BMD (g/cm^2^)∆ | 0.046 ± 0.058 | 0.072 ± 0.099 | 0.035 ± 0.042 | ns |
| T-score I | -2.25 ± 0.698 | -2.218 ± 1.068 | -2.276 ± 1.06 | ns |
| T-score II | -1.932 ± 0.762 | -1.899 ± 0.797 | -1.922 ± 0.933 | ns |
| T-score SD ∆ | 0.318 ± 0.413 | 0.368 ± 0.846 | 0.328 ± 0.443 | ns |
| Z-score I | -0.237 ± 0.68 | -0.298 ± 0.89 | -0.481 ± 1.048 | ns |
| Z-score II | 0.19 ± 0.689 | 0.207 ± 0.784 | 0.091 ± 0.873 | ns |
| Z-score SD ∆ | 0.427 ± 0.389 | 0.606 ± 0.62 | 0.525 ± 0.513 | ns |

ns - non significant, P – ANOVA P value; Tukey post-hoc test P values: *FF vs Ff; **FF vs ff; ***Ff vs ff

**Supplementary Table S8.** Delta changes in DXA parameters for the *VDR* TaqI polymorphism in L1-L4.

| **Results of DXA**  **(L1-L4)** | Genotype | | | P value  ^*^TT vs Tt  ^**^TT vs tt  ^***^Tt vs tt |
| --- | --- | --- | --- | --- |
|  | TT  *n* = 32 | Tt  *n* = 19 | tt  *n* = 11 |  |
| **BMD I** | 0.828 ± 0.144 | 0.867 ± 0.096 | 0.853 ± 0.206 | ns |
| BMD II | 0.847 ± 0.143 | 0.898 ± 0.095 | 0.936 ± 0.239 | ns |
| BMD (g/cm^2^)∆ | 0.034 ± 0.068 | 0.048 ± 0.051 | 0.068 ± 0.074 | ns |
| T-score I | -2.861 ± 1.233 | -2.596 ± 0.804 | -2.705 ± 1.711 | ns |
| T-score II | -2.539 ± 1.656 | -2.319 ± 0.753 | -2.009 ± 1.988 | ns |
| T-score SD ∆ | 0.436 ± 1.402 | 0.424 ± 0.398 | 0.574 ± 0.640 | ns |
| Z-score I | -0.948 ± 1.197 | -0.410 ± 0.935 | -0.606 ± 1.69 | ns |
| Z-score II | -0.865 ± 1.093 | -0.177 ± 0.917 | -0.031 ± 1.913 | ns |
| Z-score SD ∆ | 0.228 ± 0.653 | 0.345 ± 0.520 | 0.442 ± 0.606 | ns |

ns - non significant, *P* – ANOVA *P* value; Tukey post-hoc test *P* values: ^*^TT *vs* Tt; ^**^TT *vs* tt; ^***^Tt *vs* tt

**Supplementary Table S9.** Delta changes in DXA parameters for the *VDR* TaqI polymorphism in femoral neck.

| **Results of DXA**  **(Femoral Neck)** | Genotype | | | *P value*  ^*^TT *vs* Tt  ^**^TT *vs* tt  ^***^Tt *vs* tt |
| --- | --- | --- | --- | --- |
|  | TT  *n* = 32 | Tt  *n* = 19 | tt  *n* = 11 |  |
| **BMD I** | 0.738 ± 0.108 | 0.677 ± 0.145 | 0.729 ± 0.149 | ns |
| BMD II | 0.776 ± 0.096 | 0.773 ± 0.151 | 0.772 ± 0.117 | ns |
| BMD (g/cm^2^)∆ | 0.042 ± 0.071 | 0.089 ± 0.097 | 0.042 ± 0.059 | ns |
| T-score I | -2.125 ± 0.902 | -2.523 ± 1.009 | -2.19 ± 1.189 | ns |
| T-score II | -1.863 ± 0.691 | -1.994 ± 1.017 | -1.918 ± 0.836 | ns |
| T-score SD ∆ | 0.298 ± 0.826 | 0.476 ± 0.464 | 0.272 ± 0.489 | ns |
| Z-score I | -0.337 ± 0.795 | -0.431 ± 0.917 | -0.255 ± 1.133 | ns |
| Z-score II | 0.114 ± 0.688 | 0.293 ± 0.92 | 0.145 ± 0.794 | ns |
| Z-score SD ∆ | 0.520 ± 0.534 | 0.681 ± 0.585 | 0.400 ± 0.495 | ns |

ns - non significant, *P* – ANOVA *P* value; Tukey post-hoc test *P* values: ^*^TT *vs* Tt; ^**^TT *vs* tt; ^***^Tt *vs tt*
